# The Challenges of Distinguishing Different Causes of TMA in a Pregnant Kidney Transplant Recipient

**DOI:** 10.1155/2024/9218637

**Published:** 2024-04-27

**Authors:** A. Krelle, S. Price, M. M. Law, S. Kranz, P. Shamdasani, S. Kane, J. Unterscheider, P. Champion de Crespigny

**Affiliations:** ^1^Department of Obstetric Medicine, Royal Women's Hospital, Flemington Rd, Parkville, Victoria, Australia; ^2^Department of Nephrology, Royal Melbourne Hospital, Grattan St, Parkville, Victoria, Australia; ^3^Department of Obstetric Medicine, Frances Perry House, Flemington Rd, Parkville, Victoria, Australia; ^4^Department of Medicine, University of Melbourne, Grattan St, Parkville, VIC, Australia; ^5^Department of Diabetes and Endocrinology, Royal Melbourne Hospital, Grattan St, Parkville, Victoria, Australia; ^6^Department of Anatomical Pathology, Royal Melbourne Hospital, Grattan St, Parkville, Victoria, Australia; ^7^Department of Maternal Fetal Medicine, Royal Women's Hospital, Flemington Rd, Parkville, Victoria, Australia; ^8^Department of Obstetrics and Gynaecology, University of Melbourne, Grattan St, Parkville, Victoria, Australia

## Abstract

Thrombotic microangiopathy (TMA) reflects a syndrome of endothelial injury characterised by microangiopathic haemolytic anaemia (nonimmune), thrombocytopenia, and often end-organ dysfunction. TMA disorders are well-recognised in kidney transplant recipients, often due to an underlying genetic predisposition related to complement dysregulation, or de novo due to infection, immunosuppression toxicity, or antibody-mediated rejection. In pregnancy, TMA disorders are most commonly due to severe pre-eclampsia or HELLP, but may also be due to thrombotic thrombocytopenic purpura (TTP) or complement-mediated (atypical) haemolytic uremic syndrome (aHUS). Complement dysregulation is being recognised as playing a role in the development of preeclampsia and HELLP syndrome in addition to aHUS. Due to overlapping clinical and laboratory features, diagnosis can be difficult and delays in treatment can be life-threatening for both mother and fetus. This report describes a 32 year-old female who had two successive wanted pregnancies. The first pregnancy was terminated at 22 weeks gestation due to presumed severe preeclampsia and fetal growth restriction in the context of known chronic kidney failure due to reflux nephropathy. A living-related kidney transplant was performed to improve the chances of pregnancy resulting in a live birth. A subsequent pregnancy was complicated by progressive kidney impairment and hypertension at 22 weeks gestation. Kidney biopsy showed TMA, but the etiology was unclear. This report highlights the diagnostic dilemma of TMA in a pregnant kidney transplant recipient and a role for the anti-C5 terminal complement blockade monoclonal antibody eculizumab, in pregnancy-associated TMA, especially at a peri-viable gestation.

## 1. Introduction

Thrombotic microangiopathy (TMA) disorders are characterised by microangiopathic haemolytic anaemia (nonimmune), thrombocytopenia, and often end-organ dysfunction. Pregnancy and the post-partum period have long been recognised as high-risk periods for different forms of TMA. This report highlights the difficulty in making a diagnosis for TMA in pregnancy, especially in pregnant kidney transplant recipients with a fetus at the cusp of viability.

## 2. Clinical Case

A 30 year-old G1P0 woman with known chronic kidney failure (baseline creatinine 168 *μ*mol/L) due to reflux nephropathy conceived spontaneously and had uneventful pregnancy until 20 weeks' gestation. Hypertension, progressive kidney impairment (peak creatinine 238 *μ*mol/L), and critical fetal growth restriction (FGR) were thought to be secondary to severe early preeclampsia. She underwent a medical termination of pregnancy at 22^+1^ weeks and birthed a live male infant weighing 378 g (<1^st^ centile) with subsequent neonatal death.

To improve her chances of a live birth and because she would ultimately require a kidney transplant, she had a preemptive living-related kidney transplant when her urea, creatinine, and estimated glomerular filtration rate were 23.8 mmol/L, 327 *μ*mol/L, and 16 ml/min, respectively, and proteinuria, with a protein/creatinine ratio of 215 mg/mmol and albumin/creatinine ratio of 108 mg/mmol. This transplant occurred earlier than our usual local practice to facilitate pregnancy planning. Her immunosuppression regimen at the time of transplant was tacrolimus, mycophenolate, and prednisolone, with an induction regime consisting of methylprednisolone and basiliximab. Twenty-one months post-transplant, an acute deterioration of kidney function was found to be secondary to acute T-cell-mediated rejection. This was treated with pulsed steroids and follow-up kidney biopsy demonstrated resolution of the rejection. She underwent pre-pregnancy counselling with her nephrologist and preconception, she was changed from mycophenolate to azathioprine.

Three months later, aged 32 years, she achieved a second spontaneous pregnancy. Early antenatal history was unremarkable. Her BMI was 22.8 kg/m^2^, baseline blood pressure was 120/70 mmHg, and her creatinine was 130 *μ*mol/L. Her protein/creatinine ratio and albumin/creatinine ratio at the time were 9.3 mg/mmol and 1.1 mg/mmol, respectively. Medication included aspirin 100 mg daily, azathioprine 100 mg daily, prednisolone 5 mg daily, and tacrolimus total dose 12.5 mg daily. She was vaccinated against pneumococcal and meningococcal infections.

**Figure 1:**
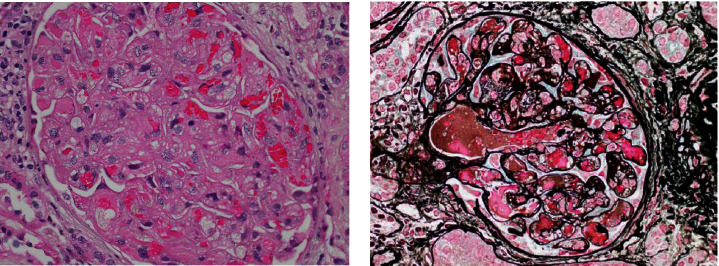
(a) H&E staining of nephron showing one glomerulus with diffuse changes of thrombotic microangiopathy with swollen endothelial cells, included capillary loops, fibrin thrombi, and fragmented erythrocytes. (b) Silver staining of nephron showing marked acute thrombotic microangiopathy with strong fibrin staining in the affected glomerulus. There was light mesangial C3c, C1q, and C4d staining only.

At 20^+1^ weeks gestation, creatinine was rising (200 *μ*mol/L) with a protein/creatinine ratio of 32 mg/mmol and an albumin/creatinine ratio of 10 mg/mmol; obvious precipitants including infection and obstruction were excluded. Her blood pressure at this time was 140/90 mmHg. Tacrolimus trough level was 5.8 *μ*g/L. Kidney transplant biopsy showed new acute TMA changes in one glomerulus and one arteriole (see Figures 1(a) and 1(b)). The sFlt-1/PIGF (soluble FMS like tyrosine kinase-1/placental growth factor) ratio was elevated at 90 suggestive of evolving early-onset preeclampsia. The tacrolimus dose was reduced and labetalol 100 mg bd was commenced. Creatinine improved to 159 *μ*mol/L. A detailed fetal anomaly scan at 21^+3^ weeks showed a normally formed, well grown fetus.

**Figure 2:**
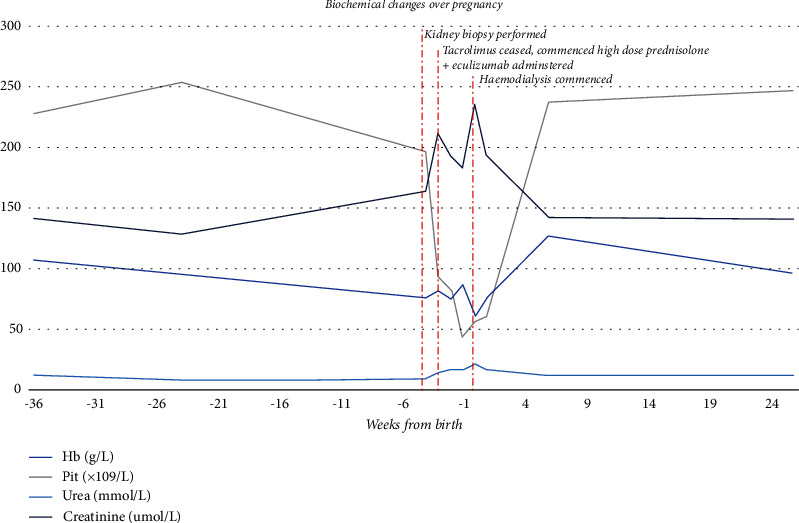
Biochemical changes before, during, and after pregnancy.

At 22^+3^ weeks, she was admitted for presumed preeclampsia or HELLP (haemolysis, elevated liver enzymes, and low platelets). Blood pressure was 135/95 mmHg with sustained clonus. Investigations showed haemoglobin 86 g/L, platelets 131 × 10^9^/L, creatinine 180 *μ*mol/L, haptoglobin <0.1 g/L and LDH 370U/L (see Figure 2). Her protein/creatinine ratio and albumin creatinine ratios at this time had significantly risen to 378 mg/mmol and 203 mg/mmol, respectively. However, the picture was not entirely consistent as her blood pressure and liver function tests remained stable, sFlt-1/PlGF was down-trending at 70, and there was no evidence of fetal growth restriction. Alternative diagnoses were considered including TMA due to tacrolimus, atypical haemolytic uremic syndrome (aHUS), and atypical PET (preeclampsia).

At 22^+5^ weeks, progressive hypertension (160/110 mmHg), thrombocytopenia (platelets 93 × 10^9^/L) and kidney impairment were noted (creatinine 206 *μ*mol/L). Serum ADAMTS13 activity was normal. Antiphospholipid antibodies were negative. At this gestation, every effort was made to prolong the pregnancy. Following a multidisciplinary team meeting, treatment with eculizumab (recombinant humanized monoclonal antibody against complement protein C5) and high-dose prednisolone was commenced, and tacrolimus was temporarily ceased for the remainder of the pregnancy. The eculizumab induction regime was as per treatment recommendations for aHUS of 900 mg weekly for four doses, followed by maintenance of 1200 mg at week five, then 1200 mg fortnightly thereafter.

Nocturnal hypertension remained problematic despite anti-hypertensive therapy. Kidney impairment (peak urea 20.5 mmol/L, creatinine 235 *μ*mol/L, urine protein/creatinine ratio 163 mg/mmol) was progressive. Intermittent haemodialysis was commenced at 24^+1^ weeks in an attempt to prolong gestation, given the maternal urea of 20.0 mmol/L, which at this level is recognised to be associated with poor fetal outcomes. Haemodialysis should be considered as a means of prolonging gestation when maternal urea concentration is 17−20 mmol/L (in addition to the clinical status of the mother) and the risk of this does not outweigh the risks of preterm delivery, as was in this case [1].

At 24^+4^ weeks, uncontrolled hypertension, progressive anaemia (60 g/L) and thrombocytopenia (36 × 10^9^/L) prompted delivery by classical caesarean section, resulting in the livebirth of a female infant weighing 538 g (<3^rd^ centile [2]). At 4 weeks postpartum, infant had prematurity-related sequelae and the mother was medically stable. Creatinine remained elevated at 180 umol/L, but she was not dialysis-dependent. Molecular genetic testing (massive parallel sequencing) did not identify a reportable sequence variant for complement dysregulopathy (aHUS, C3 glomerulonephritis, 12 gene panel), and hence eculizumab was ceased 3 months postpartum.

One-year post-partum, creatinine was at baseline 159 *μ*mol/L and kidney biopsy showed mild interstitial fibrosis with no evidence of rejection or TMA. Her offspring continued to require nocturnal home oxygen for chronic lung disease of prematurity and had low body weight but were otherwise making appropriate developmental gains.

## 3. Discussion

Thrombotic microangiopathies (TMAs) are life-threatening disorders characterised by microvascular thrombosis, endothelial injury, and organ ischemia [3]. Obstetric TMAs are particularly concerning and can result in both maternal and fetal death [3, 4]. This report explores the overlapping diagnostic features of pregnancy-associated TMA, the options for therapeutic intervention to prolong the pregnancy at a peri-viable gestation whilst balancing the risks of maternal morbidity.

Preeclampsia is common in pregnancies affected by maternal kidney impairment [5]. Aspirin use from the first trimester of pregnancy has contributed to a significant reduction in the severity of early PET [6]. Although hypertension and proteinuria may suggest evolving pre-eclampsia, these features are often pre-existing in women with kidney impairment [5]. Angiogenic factor imbalance, as can be measured by the Flt-1/PIGF ratio, is a tool now used to reliably to predict the development of PET [7]. This test remains accurate even when superimposed chronic kidney disease is present [7]. In this case, the Flt-1/PIGF ratio was not clinically available at the time of the first pregnancy, and a diagnosis of preeclampsia was made presumptively on clinical grounds. In the second pregnancy, the Flt-1/PIGF ratio was elevated early in pregnancy predicting the likely development of PET [5]. However, the rapidly evolving hypertension, thrombocytopenia, and kidney impairment were inconsistent with the modestly elevated and down-trending Flt-1/PIGF ratio; hence, a second diagnosis was considered.

HELLP is a variant of PET but occurs without hypertension and proteinuria in 20% of cases [3]. Women with HELLP usually have additional signs such as nausea, vomiting, and malaise in addition to laboratory signs including haemolytic anaemia, elevated lactate dehydrogenase (LDH), and low haptoglobin. In this case, the clinical picture was not in keeping with HELLP, and despite progressive uremia, she felt well until the 48 hours prior to delivery.

Pregnancy-associated TTP usually results from a severe deficiency in ADAMTS13, the specific metalloproteinase that cleaves ultra-large (UL) multimers of von Willebrand factor (VWF). In this case, TTP was excluded by the finding of normal ADAMTS13 activity [4]. This finding was important in this case given that pregnancy-onset TTP is one of the few causes of a high sFlt-1/PIGF ratio that is not due to PET [8].

Atypical HUS is a rare disease characterised by complement overactivation and is a diagnosis of exclusion [3]. Pregnancy is one of the most important acquired precipitants. The optimal management of patients with aHUS is with humanized monoclonal anti-C5 antibody [9]. Eculizumab has been the humanized monoclonal anti-C5 antibody available in Australia to date; however, as of 1^st^ January 2024 a longer-acting anti-C5 antibody (ravulizumab) is now available, with comparable safety and efficacy [10]. The safety of ravuizumab in pregnancy has not been established. However, as documented in multiple case series, eculizumab appears safe for mother and baby and is associated with kidney and haematological remission [3]. In this case, aHUS could not be excluded. Consistent with current literature, the decision was made to commence treatment with eculizumab on these grounds [4, 11]. Of note, there is evidence that in addition to aHUS, dysregulation of the complement system, particularly at the placental level, likely plays a role in the pathogenesis of pre-eclampsia. As such, it is possible that C5 inhibition may potentially have a role is also treating preeclampsia itself [12–14].

Around 10% of all TMAs are drug-induced and these are indistinguishable from other forms of TMA [15]. Immunosuppressive agents such as tacrolimus are classic drugs to be associated with drug induced TMA, and there are few studies to guide dosing in pregnancy. Tacrolimus has a narrow therapeutic window, and multiple factors increase the fraction of unbound tacrolimus in pregnancy such as low albumin and decreased red blood cell count, increasing the risk of toxicity [16]. Although the need to maintain adequate immunosuppression precludes drug cessation in most cases, in this patient tacrolimus was temporarily ceased and high dose prednisolone commenced in an effort to gain gestational age. Although there are reports of eculizumab being used in the context of drug-induced TMA to improve kidney and haematological parameters, there is sparse evidence to support this practice [3]. In addition to drug induced, other important causes of TMA in kidney transplant recipients include infection and antibody-mediated rejection.

Pregnancy-associated TMA can be a diagnostic dilemma with challenging clinical management problems at peri-viable gestation. Atypical preeclampsia, HELLP, calcineurin inhibitor toxicity, and aHUS were all potential contributors to TMA in this complex case. Anti-hypertensive medication, dose-reduction of tacrolimus, and eculizumab were all utilised to reach a viable gestation.

As shown by Chen et al., the risk of mortality and kidney failure is 4.46 and 5.97 times higher, respectively, for pregnant women who develop TMA compared to those who do not [17]. Each treatment undertaken was pursued with the intention of prolonging gestation, given the known poor short- and long-term outcomes associated with extreme prematurity <24 weeks gestation [18], while at the same time attempting to improve maternal outcomes and preserving her transplant function. Each was undertaken after extensive consultation with the patient, who even in the face of uncertainty was clear in her intention to prolong her pregnancy. Overall, the short- and long-term implications of our approach for the mother, offspring, and graft were considered by a multidisciplinary team including the patient.

## 4. Conclusion

Diagnosis of TMA disorders in pregnancy are difficult and may be multifactorial in pregnant kidney transplant recipients. This case highlights the need to consider multiple possible aetiologies and treat with a multifaceted approach where the diagnosis is unclear, especially if the goal is to prolong a pregnancy to viable gestation.

## Data Availability

The clinical data to support the findings of this study are available from the corresponding author upon request.
